# Supplementary material for: Effects of fire disturbance on species and functional compositions vary with tree sizes in a tropical dry forest
Source: PeerJ. 2022 May 10;10:e13270. doi: 10.7717/peerj.13270 (PMC9104091; doi:10.7717/peerj.13270)

Supplemental Information

Supplemental Table S1:

List of species included in this study. As n1994, n1999, n2004, n2009, and n2014 are abundances in year 1994, 1999, 2004, 2009, and 2014 respectively.

**Species**	**Family**	**n1994**	**n1999**	**n2004**	**n2009**	**n2014**
Acer oblongum	Sapindaceae	621	615	735	744	730
Acronychia pedunculata	Rutaceae	45	61	92	85	123
Aglaia lawii	Meliaceae	20	25	34	36	76
Aglaia spectabilis	Meliaceae	228	212	238	338	599
Alphonsea ventricosa	Annonaceae	1,151	1,225	1,489	1,725	2,202
Aphanamixis polystachya	Meliaceae	162	146	253	243	276
Baccaurea ramiflora	Phyllanthaceae	2,567	2,141	1,999	1,892	2,048
Cassia fistula	Fabaceae	105	109	190	194	186
Champereia manillana	Opiliaceae	159	137	132	121	122
Croton roxburghii	Euphorbiaceae	8,896	11,344	25,135	31,426	33,990
Cyathocalyx martabanicus	Annonaceae	1,016	867	797	726	712
Dalbergia cana	Fabaceae	244	232	278	272	294
Dalbergia cochinchinensis	Fabaceae	76	88	103	97	104
Diospyros ferrea	Ebenaceae	839	702	677	631	649
Diospyros variegata	Ebenaceae	1,398	1,176	1,101	1,011	990
Diospyros winitii	Ebenaceae	1,789	1,533	1,554	1,490	1,587
Drypetes hoaensis	Putranjivaceae	69	68	66	66	73
Dysoxylum grande	Meliaceae	182	153	164	203	276
Fernandoa adenophylla	Bignoniaceae	437	488	667	678	670
Garcinia speciosa	Clusiaceae	1,575	1,331	1,232	1,120	1,090

Supplemental Table S1 (continued):

Species	Family	n1994	n1999	n2004	n2009	n2014
Garuga pinnata	Burseraceae	110	91	95	98	104
Gluta obovata	Anacardiaceae	355	364	454	491	557
Harpullia cupanioides	Sapindaceae	127	127	120	115	111
Irvingia malayana	Irvingiaceae	159	151	148	145	143
Knema globularia	Myristicaceae	40	32	28	28	26
Lagerstroemia balansae	Lythraceae	118	91	97	84	90
Lagerstroemia tomentosa	Lythraceae	752	778	1,603	1,770	2,279
Lepisanthes rubiginosa	Sapindaceae	592	465	621	546	720
Lithocarpus cf. thomsonii	Fagaceae	58	63	55	47	46
Mallotus philippensis	Euphorbiaceae	732	695	1,024	1,216	1,711
Mangifera quadrifida	Anacardiaceae	74	100	128	176	188
Memecylon ovatum	Melastomataceae	186	214	234	317	452
Microcos paniculata	Malvaceae	100	111	212	228	269
Mitrephora thorelii	Annonaceae	2,211	1,804	1,691	1,548	1,563
Neolitsea obtusifolia	Lauraceae	1,016	999	1,369	1,564	2,594
Phoebe paniculata	Lauraceae	2,847	2,522	2,475	2,326	2,634
Picrasma javanica	Simaroubaceae	48	34	33	34	35
Polyalthia viridis	Annonaceae	5,864	5,293	5,280	4,932	5,267
Prunus arborea	Rosaceae	90	82	69	58	61
Pterospermum grandiflorum	Malvaceae	719	933	2,128	2,067	3,932
Radermachera ignea	Bignoniaceae	913	786	803	754	745
Saccopetalum lineatum	Annonaceae	1,850	1,664	1,628	1,606	2,000
Semecarpus albescens	Anacardiaceae	124	147	265	307	349
Syzygium cumini	Myrtaceae	78	104	186	221	290
Syzygium megacarpum	Myrtaceae	61	49	51	50	50

Supplemental Table S1 (continued):

**Species**	**Family**	**n1994**	**n1999**	**n2004**	**n2009**	**n2014**
Syzygium syzygioides	Myrtaceae	257	244	247	241	288
Vitex peduncularis	Lamiaceae	151	154	165	191	233
Xanthophyllum flavescens	Polygalaceae	193	184	205	242	316
Xylosma longifolium	Salicaceae	98	93	87	90	105

Supplemental Table S2:

Surface fire history in the 50-ha Huai Kha Khaeng Forest Dynamics Plot, Thailand.

Census year	Latest fire	Time since latest fire
1994	1992	2 years
1999	1998	1 year
2004	1998	6 years
2009	2005	4 years
2014	2005	9 years

Supplemental Figure S1:

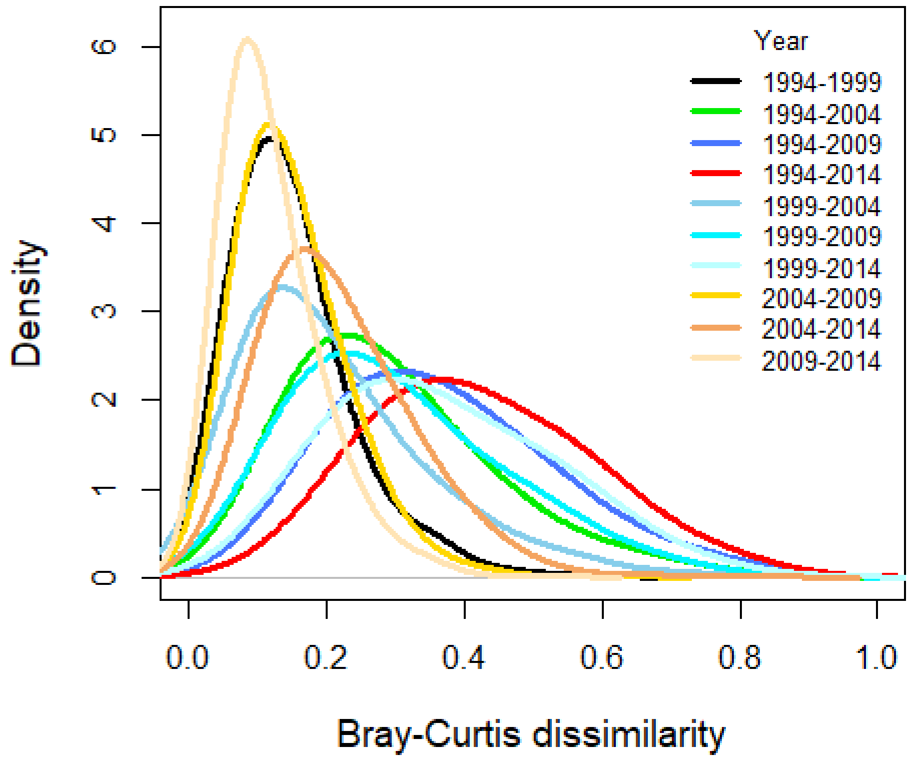

Kernel density estimates of Bray-Curtis dissimilarity for community-wide temporal turnover in species composition for ten different time intervals for all trees. The dissimilarity values were calculated at 1250 20 m x 20 m quadrats in the 50-ha Huai Kha Khaeng Forest Dynamics Plot, Thailand. Line colors represent time intervals. Increasing dissimilarity values indicates increasing differences in species composition through time.

Supplemental Figure S2:

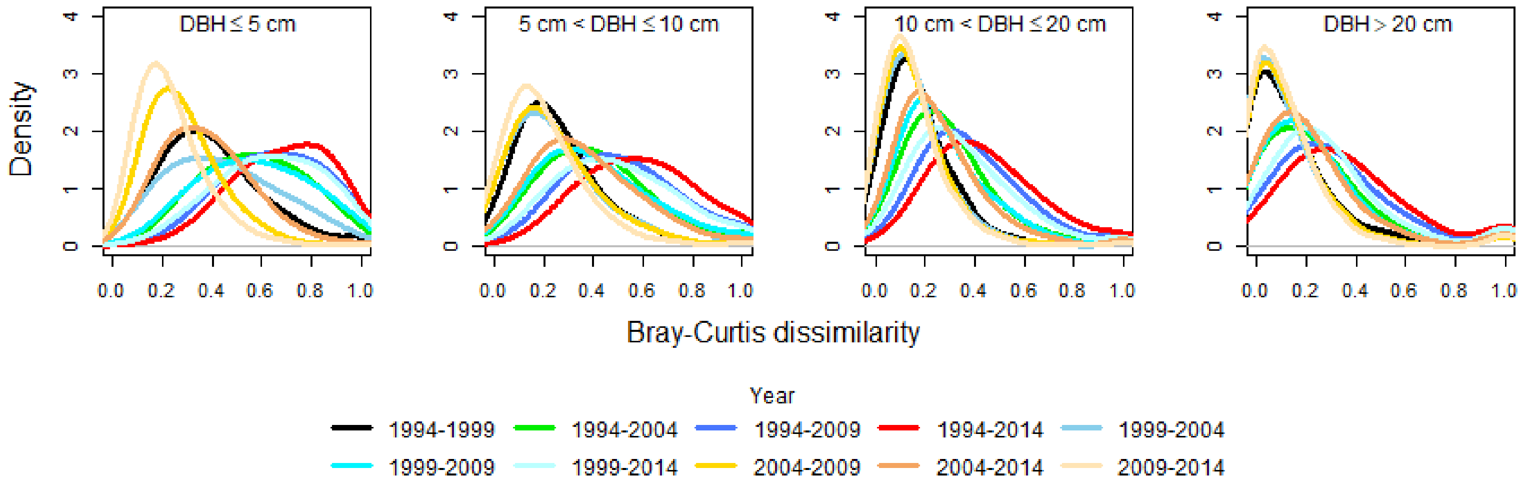

Kernel density estimates of Bray-Curtis dissimilarity for temporal turnover in species composition for ten different time intervals according to the tree diameter size classes. The dissimilarity values were calculated at 1250 20 m x 20 m quadrats in the 50-ha Huai Kha Khaeng Forest Dynamics Plot, Thailand. Line colors represent time intervals.

Supplemental Figure S3:

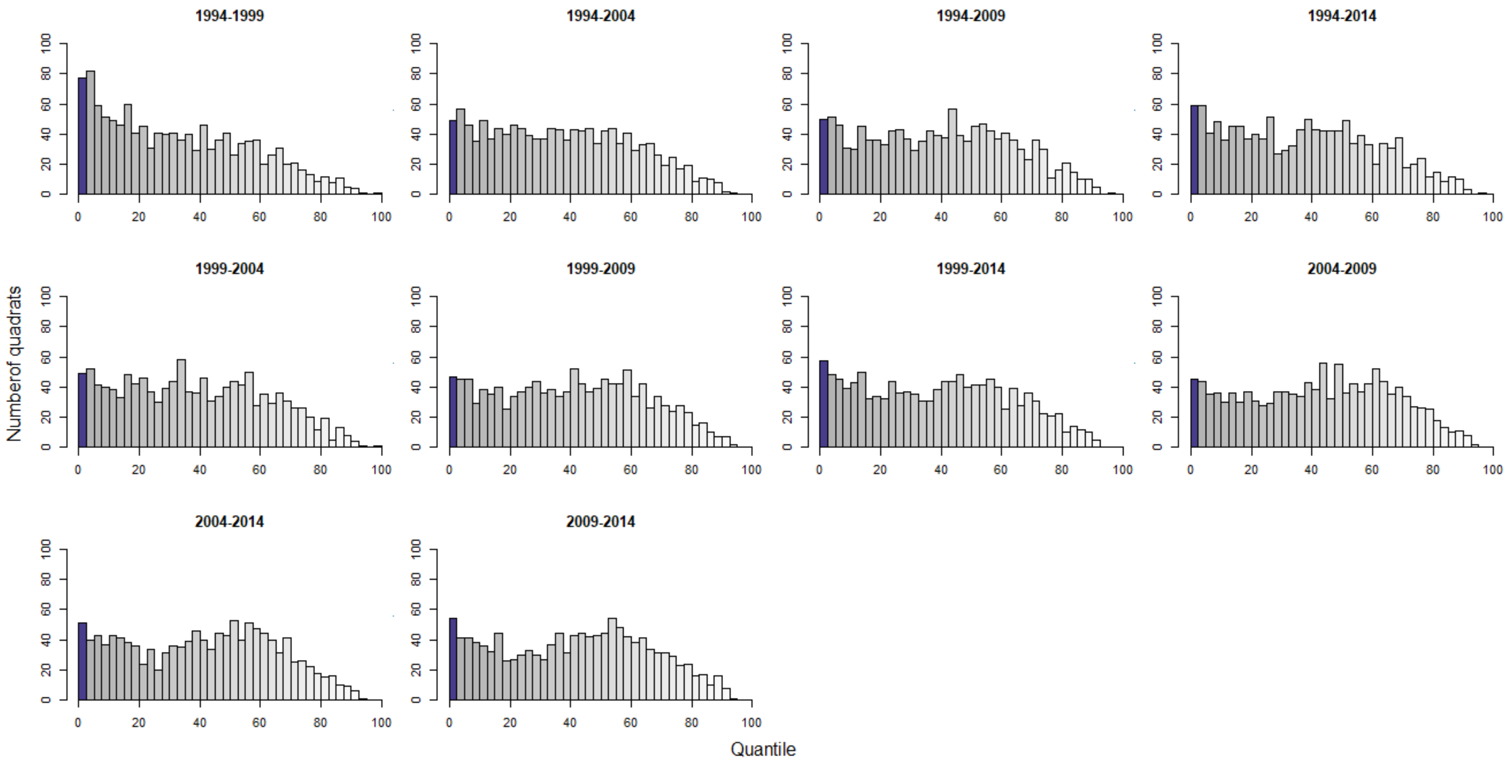

Histograms show quantile scores of functional pairwise dissimilarity for community-wide turnover in functional composition for ten different time intervals at 1250 20 m x 20 m quadrats in the 50-ha Huai Kha Khaeng Forest Dynamics Plot, Thailand.

Quantile scores represent values of significantly smaller or higher than expected by null models at 2.5 (blue bars) or 97.5 (yellow bars) respectively, at the 0.05 level. The other bars in greyscale represent the values between 2.5 and 97.5 (low values in grey and high values in white). Each bar corresponds to an interval of 2.5.

Supplemental Figure S4:

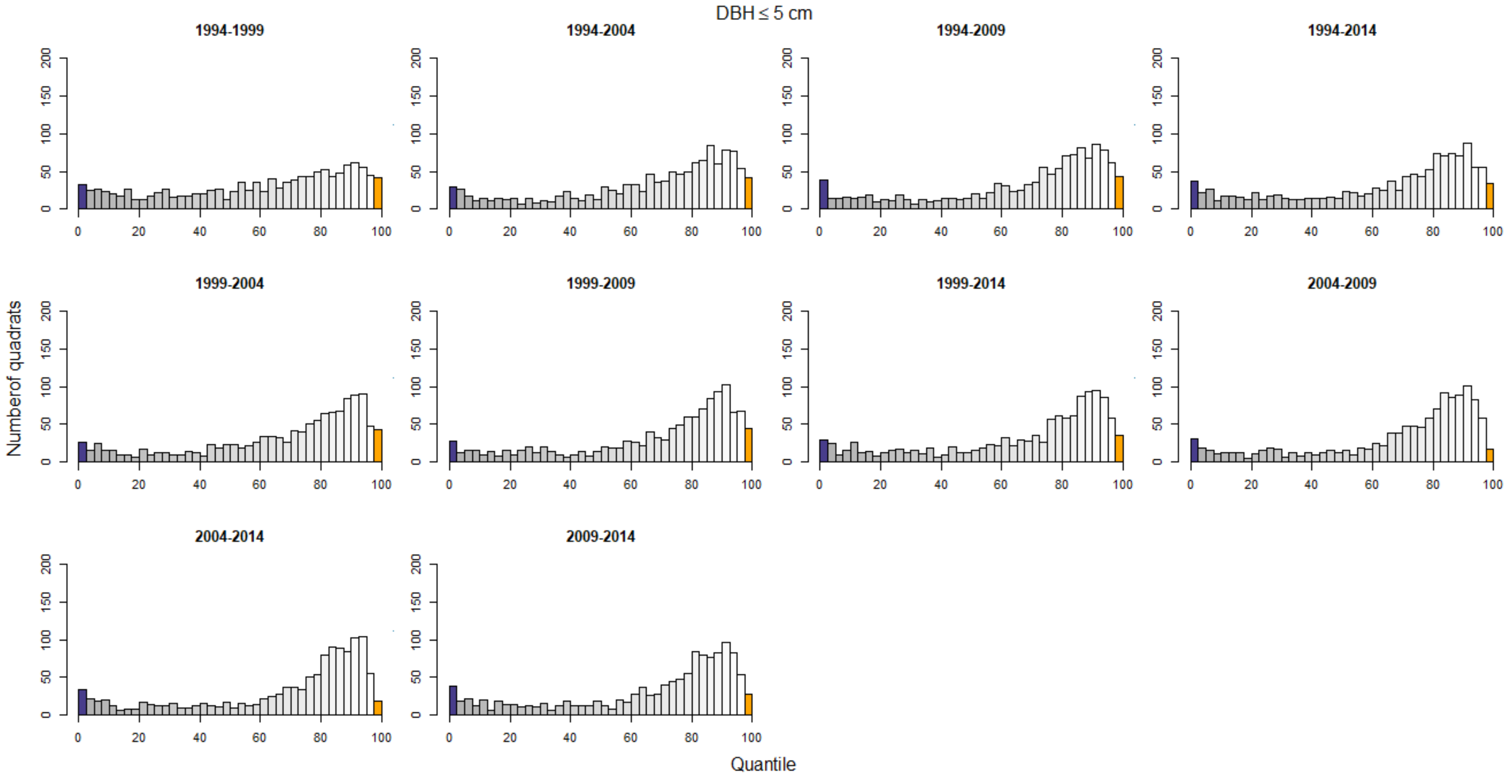

Histograms show quantile scores of functional pairwise dissimilarity for turnover in functional composition for ten different time intervals and diameter classes at 1250 20 m x 20 m quadrats in the 50-ha Huai Kha Khaeng Forest Dynamics Plot, Thailand. Quantile scores represent values of significantly smaller or higher than expected by null models at 2.5 (blue bars) or 97.5 (yellow bars) respectively, at the 0.05 level. The other bars in greyscale represent the values between 2.5 and 97.5 (low values in grey and high values in white). Each bar corresponds to an interval of 2.5.

Supplemental Figure S4 (continued):

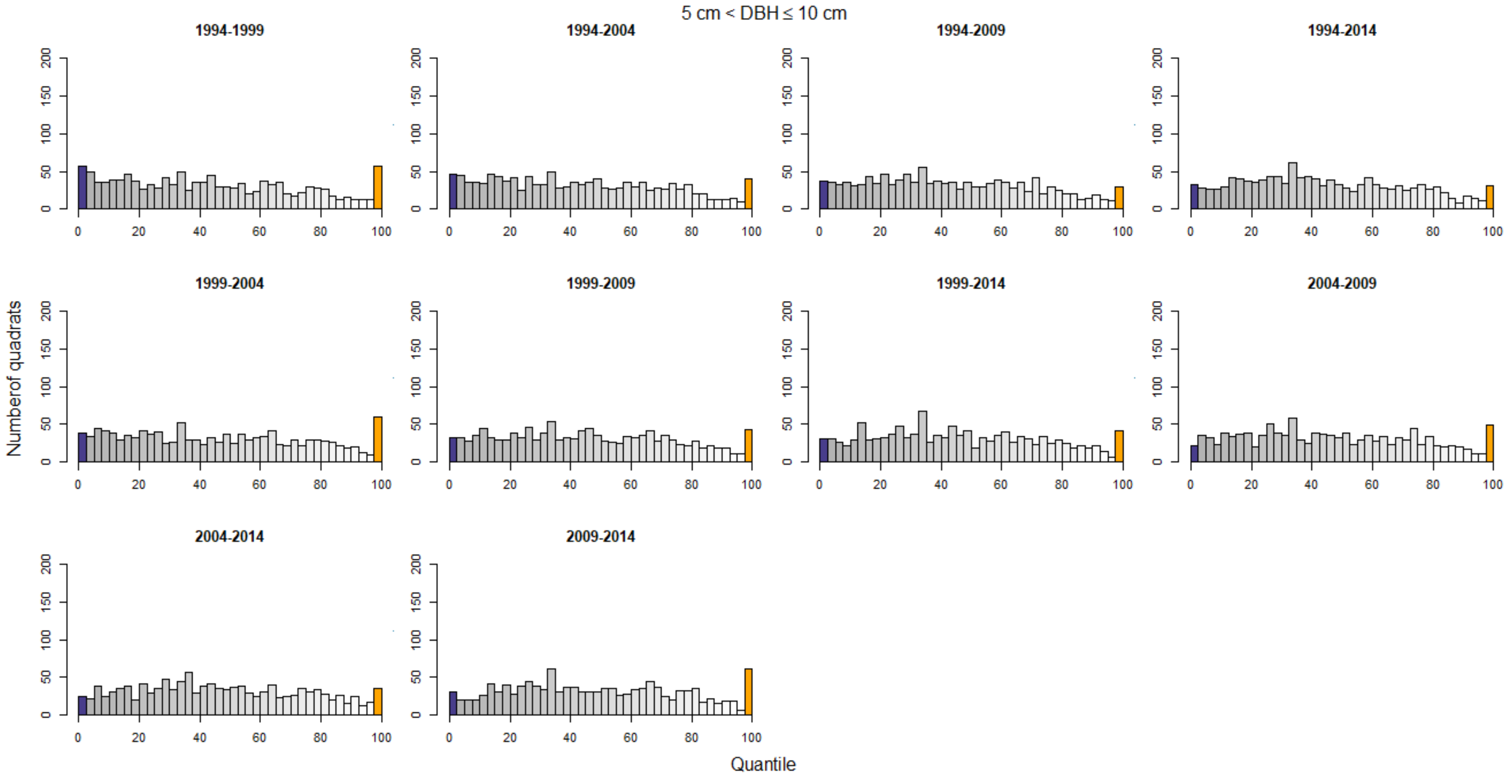

Supplemental Figure S4 (continued):

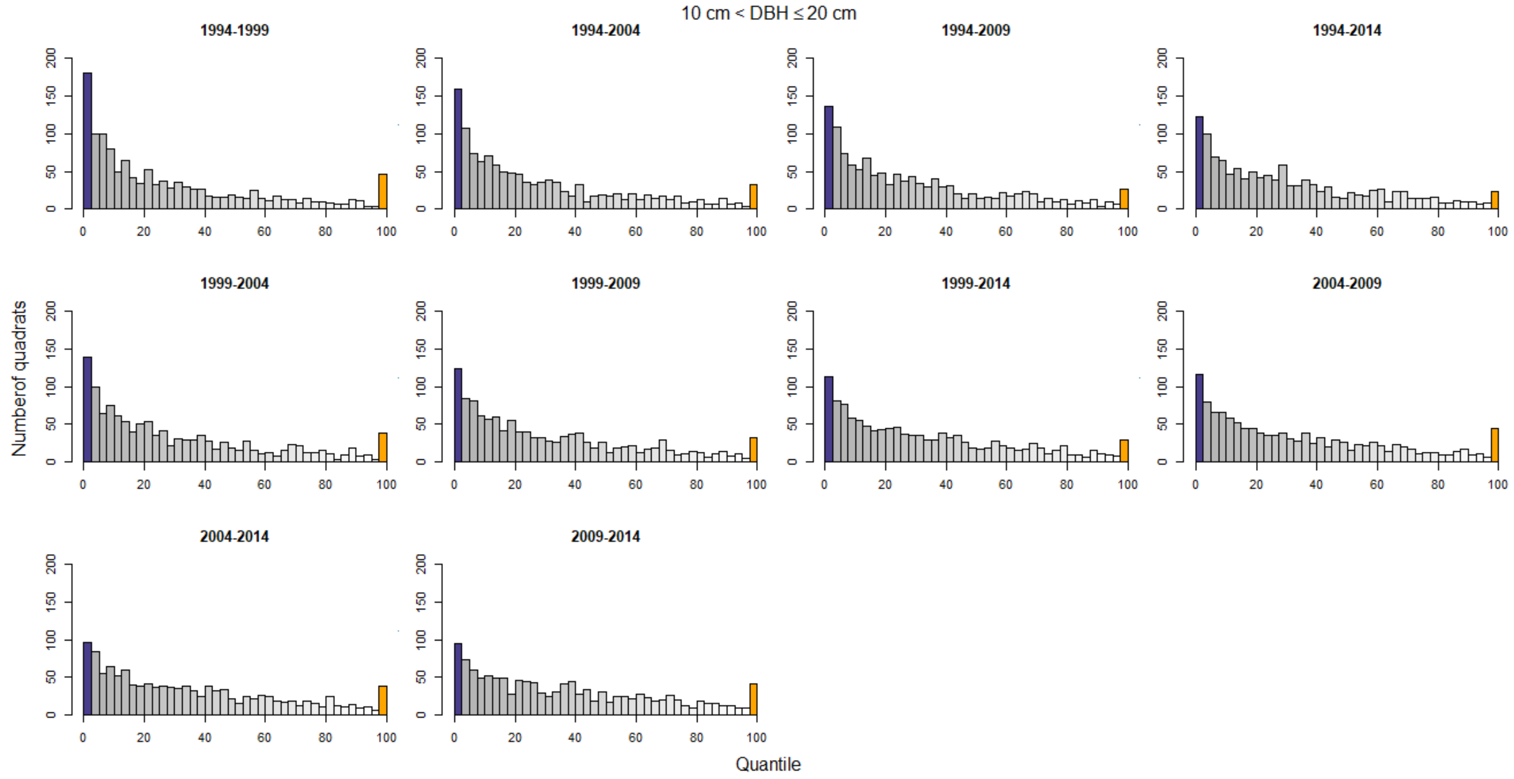

Supplemental Figure S4 (continued):

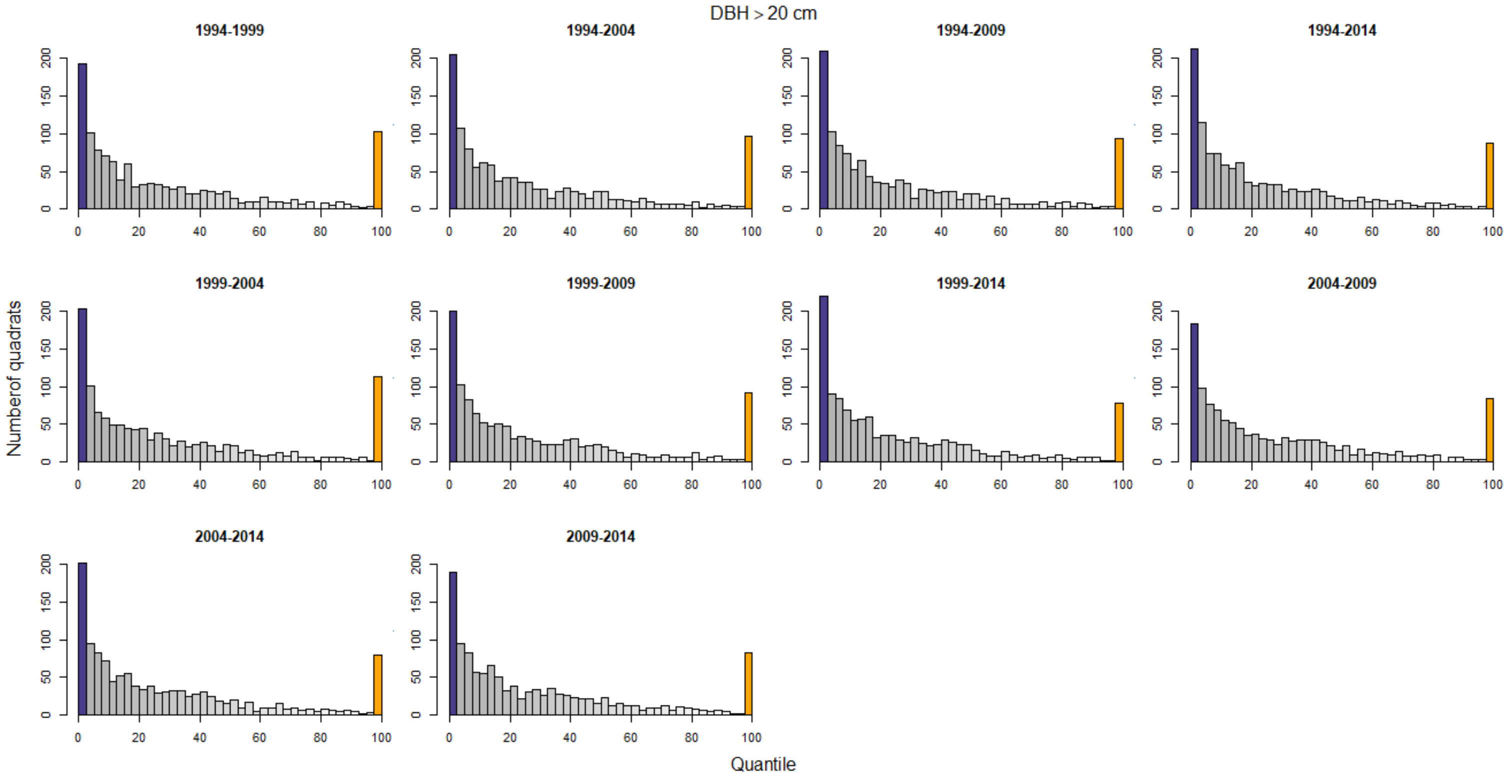

Supplement: Supplemental Information 1 [file peerj-10-13270-s001.pdf]
